# Are unrefreshing naps associated with nocturnal sleep architecture specificities in idiopathic hypersomnia?

**DOI:** 10.1093/sleep/zsad175

**Published:** 2023-07-01

**Authors:** Samantha Mombelli, Anne-Sophie Deshaies-Rugama, Hélène Blais, Zoran Sekerovic, Cynthia Thompson, Alex Desautels, Jacques Montplaisir, Milan Nigam, Julie Carrier, Nadia Gosselin

**Affiliations:** Center for Advanced Research in Sleep Medicine, Research center of the Centre intégré universitaire de santé et de services sociaux du Nord de l’Île-de-Montréal (Hôpital du Sacré-Cœur de Montréal), Montreal, Canada; Department of Psychiatry and Addictology, Université de Montréal, Montreal, Canada; Center for Advanced Research in Sleep Medicine, Research center of the Centre intégré universitaire de santé et de services sociaux du Nord de l’Île-de-Montréal (Hôpital du Sacré-Cœur de Montréal), Montreal, Canada; Department of Psychology, Université de Montréal, Montreal, Canada; Center for Advanced Research in Sleep Medicine, Research center of the Centre intégré universitaire de santé et de services sociaux du Nord de l’Île-de-Montréal (Hôpital du Sacré-Cœur de Montréal), Montreal, Canada; Center for Advanced Research in Sleep Medicine, Research center of the Centre intégré universitaire de santé et de services sociaux du Nord de l’Île-de-Montréal (Hôpital du Sacré-Cœur de Montréal), Montreal, Canada; Center for Advanced Research in Sleep Medicine, Research center of the Centre intégré universitaire de santé et de services sociaux du Nord de l’Île-de-Montréal (Hôpital du Sacré-Cœur de Montréal), Montreal, Canada; Center for Advanced Research in Sleep Medicine, Research center of the Centre intégré universitaire de santé et de services sociaux du Nord de l’Île-de-Montréal (Hôpital du Sacré-Cœur de Montréal), Montreal, Canada; Department of Neuroscience, Université de Montréal, Montreal, Canada; Center for Advanced Research in Sleep Medicine, Research center of the Centre intégré universitaire de santé et de services sociaux du Nord de l’Île-de-Montréal (Hôpital du Sacré-Cœur de Montréal), Montreal, Canada; Department of Psychiatry and Addictology, Université de Montréal, Montreal, Canada; Center for Advanced Research in Sleep Medicine, Research center of the Centre intégré universitaire de santé et de services sociaux du Nord de l’Île-de-Montréal (Hôpital du Sacré-Cœur de Montréal), Montreal, Canada; Department of Neuroscience, Université de Montréal, Montreal, Canada; Center for Advanced Research in Sleep Medicine, Research center of the Centre intégré universitaire de santé et de services sociaux du Nord de l’Île-de-Montréal (Hôpital du Sacré-Cœur de Montréal), Montreal, Canada; Department of Psychology, Université de Montréal, Montreal, Canada; Center for Advanced Research in Sleep Medicine, Research center of the Centre intégré universitaire de santé et de services sociaux du Nord de l’Île-de-Montréal (Hôpital du Sacré-Cœur de Montréal), Montreal, Canada; Department of Psychology, Université de Montréal, Montreal, Canada

**Keywords:** idiopathic hypersomnia, naps, phenotype subtypes, sleep architecture

## Abstract

**Study Objectives:**

Unrefreshing naps are supportive clinical features of idiopathic hypersomnia (IH) and are reported by more than 50% of IH patients. They are, however, not mandatory for the diagnosis, and their pathophysiological nature is not understood. This study aimed at verifying whether IH patients with and without unrefreshing naps constitute two subtypes of IH based on their demographic/clinical characteristics, and sleep architecture.

**Methods:**

One hundred twelve IH patients underwent a polysomnography (PSG) followed by a multiple sleep latency test (MSLT). They completed questionnaires on excessive daytime sleepiness, mood, and sleep quality. They were met by sleep medicine physicians who conducted a semi-structured clinical interview and questioned them on refreshing aspects of their naps. Patients who reported unrefreshing naps were compared to patients reporting refreshing naps on questionnaires, MSLT and PSG variables, with age as a covariable. As sensitivity analyses, we performed the same comparisons in participants presenting objective markers of IH and those diagnosed with IH based only on clinical judgment (subjective IH), separately.

**Results:**

In the whole sample, 61% of patients reported unrefreshing naps. These participants had less awakenings, a lower percentage of N1 sleep, less sleep stage transitions, and a higher percentage of REM sleep on the nighttime PSG compared to the refreshing naps subgroup. When subjective and objective IH patients were tested separately, more group differences were observed on PSG for subjective IH patients.

**Conclusions:**

Patients with unrefreshing naps have less fragmented sleep compared to those with refreshing naps. Future studies should investigate whether this group difference indicates a weaker arousal drive.

## Graphical Abstract

**Figure:**
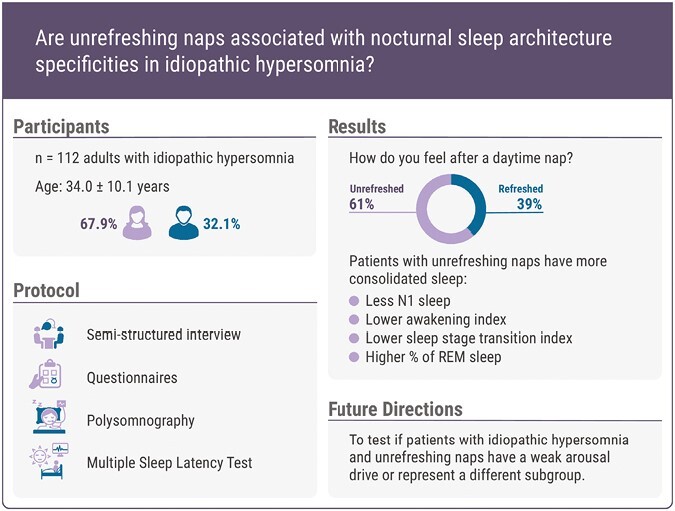

**Statement of Significance**

Idiopathic hypersomnia (IH) is a disorder characterized by excessive daytime sleepiness and it has been observed that more than 50% of IH patients report unrefreshing naps. Mechanisms underlying unrefreshing naps remain uncertain and whether patients with and without unrefreshing naps constitute two subtypes of IH is unknown. Here, we compared clinical profiles and sleep architecture of a large cohort of IH patients with and without unrefreshing naps. We showed that IH patients reporting unrefreshing naps had more consolidated sleep compared to patients with refreshing naps. These results suggest that unrefreshing naps are not due to poor sleep quality, but could rather be explained by a weak arousal drive. More studies are needed to investigate the multifactorial nature of IH and better understand subgroup differences in IH patients.

## Introduction

Idiopathic hypersomnia (IH) is a central disorder of hypersomnolence characterized by chronic excessive daytime sleepiness despite normal or prolonged sleep [1]. Individuals with IH often report long and unrefreshing naps as well as sleep drunkenness, which is an excessive form of sleep inertia [2, 3]. IH occurs in the absence of cataplexy and its pathophysiology remains poorly understood as no clear biomarkers have been identified. IH most often begins during young adulthood and women represent approximately 60% of cases [4–6]. Patients have significant IH-related disabilities, most typically related to driving, work and school, and family and social life [7].

A unique aspect of IH is the paradox between the irrepressible need to take naps and the feeling that they are, most of the time, nonrestorative or unrefreshing. In fact, in a study performed on 77 IH patients, 76 patients had daytime naps more than 5 days a week, 87% of naps lasted for more than 60 min, but 78% of patients described them as unrefreshing [2]. Similar results were also found in other studies [4, 5, 8, 9]. Interestingly, unrefreshing naps are not commonly observed in other hypersomnolence disorders, such as narcolepsy, and are to date considered as an additional supportive clinical feature for IH diagnosis [1]. However, as recently proposed, current ICSD-3 diagnostic criteria for IH need to be revised and the quality of naps could be proposed as a diagnostic criterion [3].

The mechanisms underlying the unrefreshing quality of naps in IH remain uncertain. One study using self-reports in IH patients and healthy controls showed that for long naps (defined as > 30 min), 52% of IH patients and 65% of healthy controls reported unrefreshing naps. However, for short naps, IH patients were more susceptible to report unrefreshing naps compared to controls (75% vs. 35% for IH and controls, respectively) [9]. Nap duration, therefore, seems associated with their unrefreshing aspects for healthy controls in whom longer naps, with the possible occurrence of N3 sleep, could lead to sleep inertia and the feeling of naps being unrefreshing. However, in IH patients, nap duration is not a factor associated with the refreshing aspects of sleep. This important observation would need replication, as it has the potential to deepen our understanding of IH pathophysiology. Other factors could be associated with the unrefreshing aspects of naps, such as IH severity measured with total sleep time on 24 hours, mean sleep onset latency on the multiple sleep latency test (MSLT) and subjective excessive daytime sleepiness (EDS), use of psychoactive medications altering sleep, and psychiatric comorbidities. However, no studies have specifically investigated those factors. Finally, whether patients with and without unrefreshing naps constitute two IH subtypes is also unknown. The objectives of this study were thus to characterize the clinical profile and sleep architecture of IH patients with and without unrefreshing naps. We also aimed to test whether these group differences were observed in both patients presenting objective markers of IH (i.e. total 24-hour sleep time ≥ 660 min or MSLT ≤ 8 min, “objective IH”) and in IH patients diagnosed based on clinical features and complaints without reaching objective markers cut-offs (“subjective IH”).

## Methods

### Subjects

All participants included in the present study have visited the sleep clinic of the Center for Advanced Research in Sleep Medicine at the *Hôpital du Sacré-Coeur de Montréal* (*Centre intégré universitaire de santé et services sociaux du Nord-de-l’Ile-de-Montréal*, Montreal, Canada) between 2000 and 2019. They were all referred to the sleep clinic for suspected IH or unexplained EDS. Participants were included if they were aged between 18 and 60 years old and had a diagnosis of IH made by a sleep physician based on the clinical interview and a full night of in-laboratory polysomnography (PSG), followed by a MSLT. Diagnoses were revised according to the current criteria [1], which include: 1) daily periods of irrepressible need to sleep or daytime lapses into sleep occurring for at least 3 months; 2) no cataplexy; 3) < 2 sleep onset in rapid-eye movement periods (SOREMPs) on the PSG-MSLT procedure; 4) mean sleep onset latency ≤ 8 min on the MSLT, or mean total 24-hour sleep time ≥ 660 min on 24-hour PSG monitoring or by wrist actigraphy in association with a sleep log (averaged over at least seven days with unrestricted sleep). Nearly half of the participants (53/112) met the criteria for IH but failed to reach mean sleep onset latency ≤ 8 min on the MSLT. Most patients did not perform an ad libitum PSG or had available actigraphy. They could still be diagnosed with IH based on clinical judgment of the sleep physician, especially when they reliably reported a long ad libitum sleep duration and sleep drunkenness, as recommended by the ICSD-3 notes and as previously performed [2, 8]. They were identified as having “subjective IH” in the present study. Exclusion criteria were: 1) change in sleep disorder diagnosis over time; 2) apnea-hypopnea index ≥ 15; 3) neurologic comorbidities (e.g. Parkinson’s disease, epilepsy, multiple sclerosis, moderate to severe traumatic brain injury, dementia, stroke); 4) shift work; 5) circadian rhythm disorders, chronic sleep deprivation or other sleep disorders (e.g. somnambulism, restless leg syndrome, rapid-eye movement (REM) sleep behavior disorder, insomnia); 6) medical conditions or medications that could explain hypersomnolence; 7) major psychiatric disorders (e.g. schizophrenia, bipolar disorder); 8) use of psychoactive and antidepressant medication that could not be stopped for the PSG recording. This study was approved by the *Centre intégré universitaire de santé et services sociaux du Nord-de-l’Ile-de-Montréal* Research Ethics Board (REB 2020-1905) and was performed in accordance with the Declaration of Helsinki.

### Clinical interviews and questionnaires

All participants underwent a semistructured clinical interview during which a sleep medicine physician completed a comprehensive medical history, including all current and past diseases and medication, as well as those which could have any residual effects on the patient’s sleep. A detailed sleep history was considered, including: sleep schedule and hygiene; associated nocturnal or diurnal symptoms; daytime functioning; nature, severity, timing and circumstances of the sleep complaints; and clinical data supporting an IH diagnosis, such as subjective daytime sleepiness, number and duration of naps. This interview included the question: “In general, do you feel that your daytime naps are refreshing or unrefreshing?,” or “In general, do you feel refreshed or unrefreshed after a daytime nap?.” Following this interview, the physician indicated in the clinical report whether the naps were refreshing or not. Participants were asked to fill out a sleep diary for two weeks before the PSG, as well as the following self-administered questionnaires: Beck Depression Inventory (BDI-II) [10], categorizing the severity of depressive symptoms as minimal (score: 0–13), mild (score: 14–19), moderate (score: 20–28), or severe (score: 29–63); Beck Anxiety Inventory (BAI) [11], categorizing the severity of anxiety symptoms as minimal (score: 0–7), mild (score: 8–15), moderate (score: 16–25), or severe (score: 30–63); Epworth Sleepiness Scale (ESS) [12], assessing daytime sleepiness through the participant’s self-reported sleep propensity during everyday life (score: 0–24, higher scores indicating greater daytime sleepiness); and a customized questionnaire administered the morning after the PSG asking whether their nighttime sleep was restorative or not (“Do you feel rested this morning?”).

### Overnight PSG and MSLT

Psychoactive and antidepressant medication was stopped at least 5 half-lives before the PSG recording. In the laboratory, bedtime and wake time were determined according to the participant’s usual schedule, but did not occur before 22:00 (bedtime) and later than 07:00 (wake time). PSG (Harmonie Stellate Systems, Montreal, Canada) included at least 4 electroencephalographic (EEG) electrodes (C3, C4, O1, and O2), and PSG recorded after 2013 included the following electrodes: F3, F4, C3, C4, O1, and O2, linked to contralateral earlobes with 10 kΩ resistance, as well as electrooculogram, submental electromyogram, electrocardiogram, bilateral anterior tibialis electromyogram, abdominal strain gauge, oronasal canula, video and audio recordings, and transcutaneous finger pulse oximeter. An adapted version of the MSLT with four naps (09:00, 11:00, 13:00, and 15:00) was performed the day after [13], where only the EEG (same electrodes as for the overnight PSG), electrooculogram, and submental electromyogram were kept in place.

### Data analyses

PSG and MSLT were scored on 30 s epochs by experienced medical electrophysiology technologists, according to current criteria [13, 14]. SOREMP was defined as the occurrence of a REM period within 15 min after the onset of sleep during overnight PSG as well as in the MSLT. For the MSLT, the average sleep latency and total sleep time were measured for each participant.

For each sleep recording, we measured the following variables: total sleep time, sleep onset latency, REM sleep onset latency, sleep efficiency, wake after sleep onset, duration and percentage of each sleep stage (wake, N1, N2, N3, total NREM sleep, and REM sleep), apnea-hypopnea index (number of breathing events/hour; apneas = reduction of airflow ≥ 90% for at least 10 s, hypopneas = diminution of airflow ≥ 30% for at least 10 s, ending with an oxygen desaturation ≥ 3% or with an arousal [14]), awakening index (number of awake epochs/hour), microarousal index (number/hour) and periodic legs movement during sleep index (number/hour). We also measured the number of transitions from one stage (awake, N1, N2, N3, and REM) to any other stage throughout the night and a sleep stage transition index was calculated (number of sleep stage transitions/hour).

### Statistical analyses

To characterize the clinical profile and sleep architecture of IH patients with and without unrefreshing naps, Groups (unrefreshing vs refreshing naps) were compared on continuous variables using ANCOVAs adjusted for age with Group as the independent variable and demographic, clinical and MSLT/PSG characteristics as dependent variables. Chi-square tests were used to analyze differences between Groups on dichotomous variables. Nighttime sleep was divided into i) thirds (same number of sleep epochs for each third; for a number of epochs not divisible by three, the first third followed by the second third could contain one sleep epoch more than the last third), and ii) halves. We also compared the proportion of participants reporting unrefreshing/refreshing naps based on the refreshing aspect of their nighttime sleep using chi-square tests, and their PSG and MSLT variables using ANCOVAs controlled for age.

We performed sensitivity analyses to evaluate whether the quality of naps (refreshing vs unrefreshing) was similar in objective and subjective IH. For these two subgroups separately, we performed ANCOVAs adjusted for age with Group as an independent factor and sociodemographic/clinical, MSLT, and PSG variables as dependent variables.

Finally, to help the reader fully understand our results, we performed the same analyses (ANCOVAs adjusted for age) to evaluate how patients with objective IH differ from those with subjective IH for demographic, clinical, and MSLT/PSG variables (see Supplementary Material).

Data were reported as means and standard deviations (continuous variables), and proportions and percentages (categorical variables). Significance levels were set at *p* < .05. All analyses were performed using SPSS 22 and JASP version 0.16.2.

## Results

### Demographic and clinical characteristics

We included 112 IH patients (34.0 ± 10.1 years old, 67.9% women), with 61% (*N* = 68) reporting unrefreshing naps. Demographic variables, clinical characteristics and statistics are presented in Table 1. The unrefreshing naps group was younger and more susceptible to report nonrestorative nighttime sleep in the morning after the PSG compared to the refreshing naps group. There were no group differences in body mass index, or depression, anxiety and EDS symptom severity.

**Table 1.** Demographic/clinical, polysomnographic and MSLT data of IH participants reporting unrefreshing vs. refreshing naps adjusted for age (when applicable)

	Unrefreshing naps (*n* = 68)	Refreshing naps (*n* = 44)	*F*(df) or χ^2^	*p*-value	Effect size or Cramer’s V
DEMOGRAPHIC/CLINICAL DATA					
Women (*n*; %)	50; 73.5%	26; 59.1%	χ^2^ = 2.553	.110	0.151
Age (years)	31.6 ± 8.4	37.7 ± 11.4	*F*_(1,110)_ = 10.395	**.002**	0.086
BMI (kg/m^2^)	23.9 ± 3.6	25.6 ± 4.8	*F*_(1,99)_ = 3.102	.081	0.030
Felt rested after the nighttime PSG (*n*; %)	6; 17.6%	13; 46.4%	χ^2^ = 5.985	**.014**	0.311
ESS score	16.5 ± 4.1	17.4 ± 2.7	*F*_(1,104)_ = 0.589	.445	0.005
Beck Depression Inventory score	10.8 ± 8.9	10.3 ± 6.5	*F*_(1,91)_ = 0.197	.659	0.002
Beck Anxiety Inventory score	8.5 ± 8.4	7.9 ± 6.3	*F*_(1,91)_ = 0.197	.659	0.002
POLYSOMNOGRAPHIC DATA					
Total sleep time (min)	461.5 ± 45.0	446.6 ± 36.7	*F*_(1,108)_ = 1.117	.280	0.010
Sleep onset latency (min)	10.3 ± 8.5	9.2 ± 8.3	*F*_(1,108)_ = 0.418	.520	0.004
REM sleep latency (min)	97.4 ± 46.2	91.5 ± 45.6	*F*_(1,108)_ = 0.066	.797	6.029e-4
WASO (min)	37.8 ± 28.1	43.4 ± 26.0	*F*_(1,108)_ = 0.003	.955	2.632e-5
Awakening index (nb./h)	3.0 ± 1.2	3.8 ± 1.6	*F*_(1,108)_ = 6.156	**.015**	0.051
Sleep efficiency (%)	92.4 ± 5.6	91.1 ± 5.5	*F*_(1,108)_ = 0.045	.883	3.688e-4
N1 (%)	8.4 ± 3.7	11.9 ± 6.7	*F*_(1,108)_ = 7.491	**.007**	0.061
N2 (%)	56.4 ± 7.4	56.6 ± 5.9	*F*_(1,108)_ = 1.002	.319	0.008
N3 (%)	14.1 ± 6.8	12.4 ± 7.7	*F*_(1,108)_ = 0.079	.779	5.603e-4
REM sleep (%)	21.1 ± 4.5	19.2 ± 3.7	*F*_(1,108)_ = 4.211	**.043**	0.037
Microarousal index	8.2 ± 4.5	10.5 ± 6.0	*F*_(1,108)_ = 3.104	.081	0.027
AHI (events/h)	1.5 ± 2.0	2.7 ± 3.4	*F*_(1,108)_ = 2.353	.128	0.020
PLMS index	6.8 ± 11.7	9.5 ± 16.1	*F*_(1,108)_ = 0.034	.854	2.922e-4
Sleep stage transitions index (nb./h)	21.9 ± 5.1	25.1 ± 6.3	*F*_(1,108)_ = 5.756	**.018**	0.049
Transition REM to other stages (nb./h)	16.0 ± 6.6	17.3 ± 5.9	*F*_(1,108)_ = 0.563	.455	0.005
MSLT DATA					
Mean Total sleep time (min)	11.5 ± 3.7	11.7 ± 3.0	*F*_(1,108)_ = 0.212	.646	0.002
Mean Sleep onset latency (min)	8.5 ± 4.7	8.2 ± 4.2	*F*_(1,108)_ = 0.007	.933	6.498e-5
Mean Sleep efficiency (%)	86.2 ± 12.8	85.6 ± 15.0	*F*_(1,108)_ = 5.836e-4	.981	5.312e-6
SOREMPs (nb.)	0.4 ± 0.6	0.2 ± 0.4	*F*_(1,108)_ = 1.426	.235	0.013

The results are expressed as the mean ± standard deviation for continuous variables, and as the number of participants and percentage for categorical variables. BMI: body mass index; ESS: Epworth Sleepiness Scale; PSG: polysomnography; AHI: apnea-hypopnea index; nb: number; NREM: non-REM sleep; PLMS: periodic legs movement in sleep; REM: rapid eye movement sleep; WASO: wake after sleep onset; SOREMPs: sleep onset in rapid eye movement period

### Sleep architecture and MSLT data

**Figure 1.**
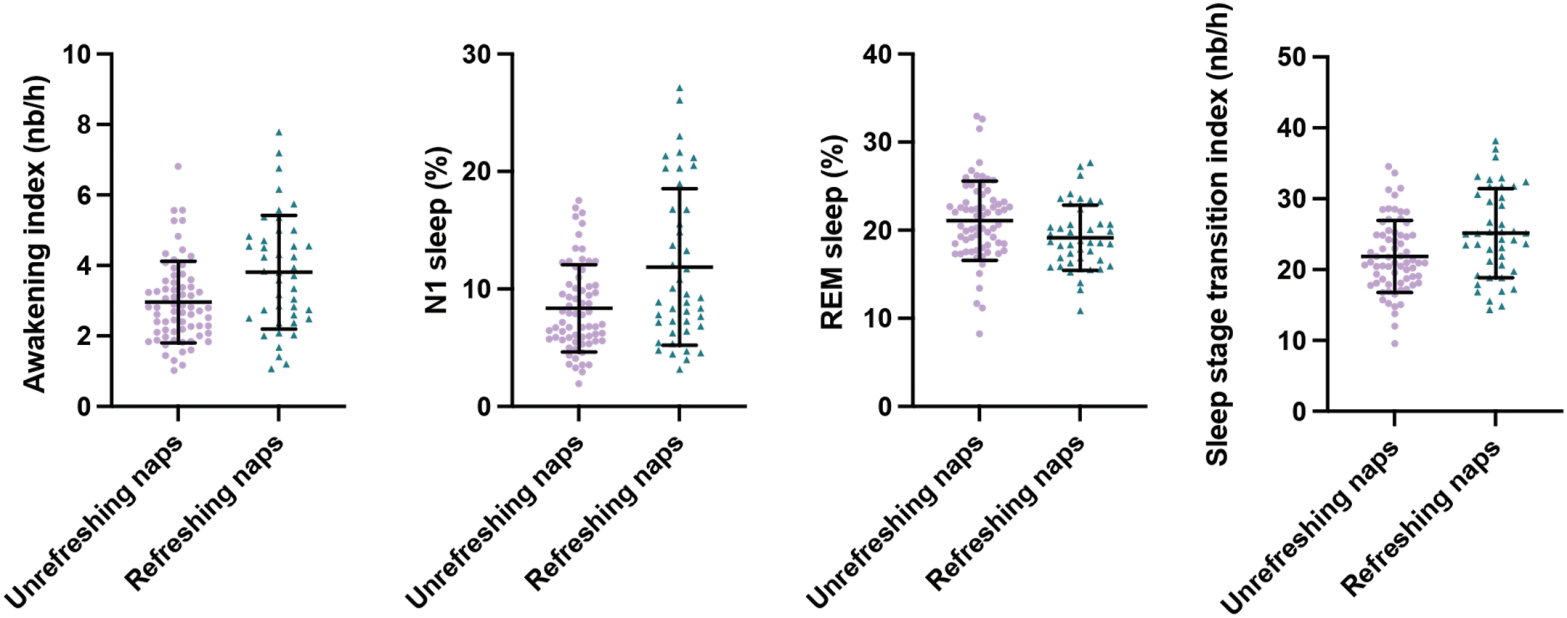
Box plots representing group differences on polysomnographic variables, adjusted for age. Each point represents one participant. The central lines in each box represent the medians, and the top and bottom of the boxes represent the 25th and 75th percentile. The whiskers represent all values not considered as outliers.

Nighttime PSG and MSLT variables and statistics for the unrefreshing and refreshing naps groups adjusted for age are presented in Table 1. Significant results are presented in Figure 1.

In the nighttime PSG, participants with unrefreshing naps had a lower awakening index and a lower percentage of N1 sleep than those reporting refreshing naps. They also showed a higher percentage of rapid eye movement sleep (REM) compared to the refreshing naps group. In terms of sleep microarchitecture, participants with unrefreshing naps had a lower sleep stage transition index compared to the refreshing naps group. When transitions were analyzed by sleep stages, the unrefreshing naps group showed less transitions from N1 to N2 and from N1 to other sleep stages, compared to the refreshing naps group. No group difference was found for microarousals.

When we explored group differences for sleep stages according to the time of the night, we found that the lower percentage of N1 sleep in the unrefreshing naps group was only observed in the last third of the night (unrefreshing naps: 3.4 ± 1.7% vs. refreshing naps: 5.0 ± 2.9%, *p* < .001). We obtained the same result when dividing the night into two halves (unrefreshing naps: 4.6 ± 2.3% vs. refreshing naps: 6.8 ± 3.7%, *p* = .003).

When we performed the analyses based on the unrefreshing/refreshing aspect of nighttime sleep, the unrefreshing nighttime group was more susceptible to report unrefreshing naps during the day compared to those reporting restorative nighttime. No group difference was found on the PSG and MSLT characteristics.

There were no significant group differences for MSLT characteristics, including mean sleep onset latency and number of naps slept. When each nap was assessed separately, no significant group difference was found.

### Sensitivity analyses

**Figure 2.**
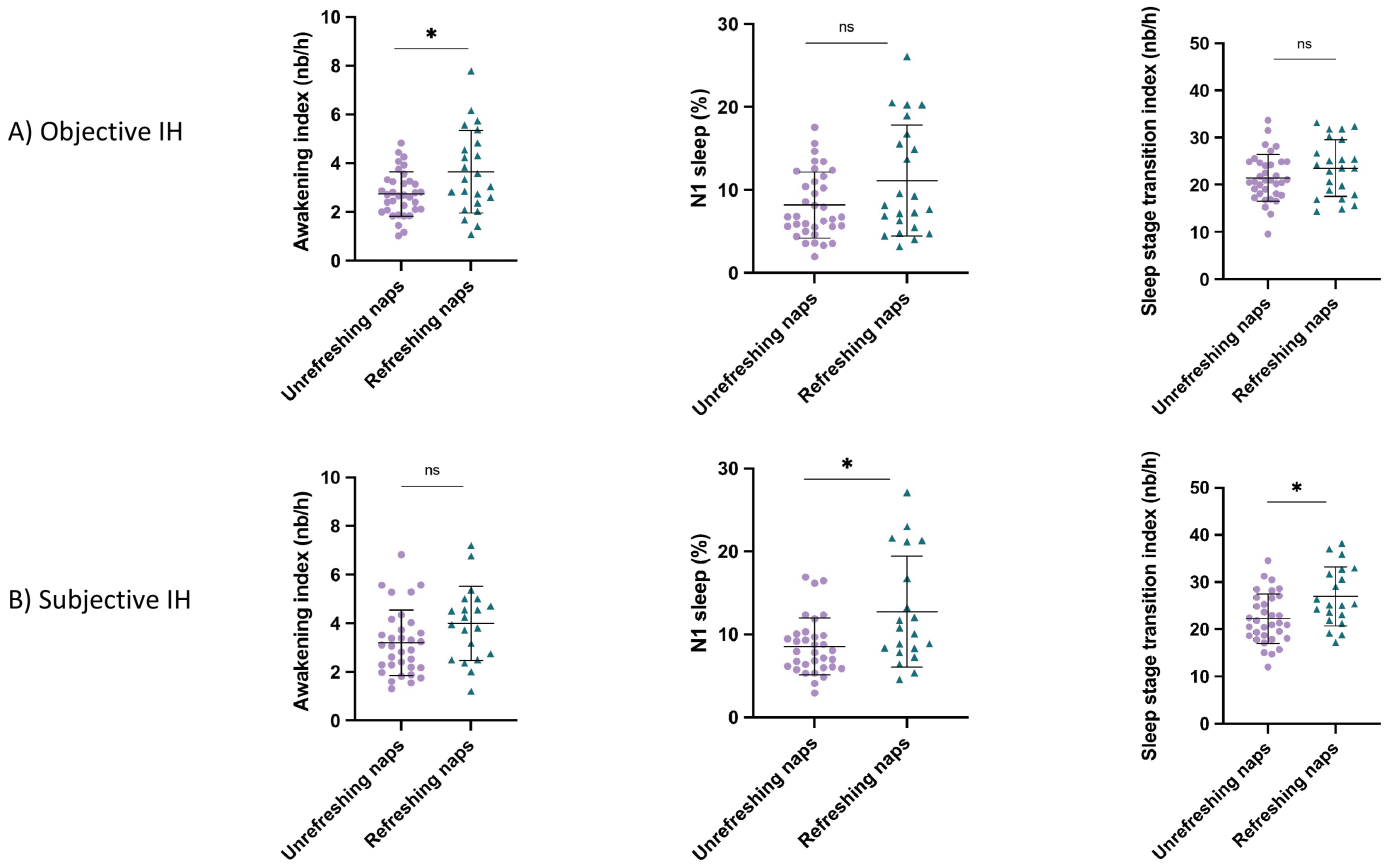
Box plots presenting the awakening index, % of N1 sleep, and sleep stage transition index in objective (top graphs) and subjective IH (bottom graphs) with or without unrefreshing naps, separately. Each point represents one participant. The central lines in each box represent the medians, and the top and bottom of the boxes represent the 25th and 75th percentile. The whiskers represent all values not considered as outliers. Statistically significant results are expressed with *. ns: not significant.

Considering that 47% of our sample (*N* = 53) was diagnosed based on clinical features and complaints, we compared those patients (“subjective IH”) (31.6 ± 8.7 years old, 66.0% women) to patients presenting objective markers of IH (“objective IH”) (36.2 ± 10.8 years old, 69.5% women). Demographic/clinical data, nighttime PSG, MSLT variables and statistics for the objective IH subgroup are presented in Table 2, while those of the subjective IH subgroup are presented in Table 3. Sleep variables that differ between objective and subjective IH are presented in Figure 2.

**Table 2.** Demographic/clinical, polysomnographic and MSLT data of participants with objective IH reporting unrefreshing vs. refreshing naps adjusted for age (when applicable)

	Unrefreshing naps (*n* = 35)	Refreshing naps (*n* = 24)	*F*(df) or χ^2^	*p*-value	Effect size or Cramer’s V
DEMOGRAPHIC/CLINICAL DATA					
Women (*n*; %)	27; 65.9%	14; 34.1%	χ^2^ = 2.376	.123	0.201
Age (years)	33.3 ± 9.6	40.4 ± 11.2	*F*_(1,57)_ = 6.845	**.011**	0.107
BMI (kg/m^2^)	24.2 ± 3.9	26.7 ± 4.9	*F*_(1,50)_ = 4.564	**.038**	0.082
Felt rested after the nighttime PSG (n; %)	2; 22.2%	7; 77.8%	χ^2^ = 3.031	.082	0.299
ESS score	16.7 ± 4.4	17.2 ± 2.6	*F*_(1,52)_ = 0.077	.783	0.001
Beck Depression Inventory score	12.1 ± 10.3	10.6 ± 7.2	*F*_(1,42)_ = 0.353	.556	0.008
Beck Anxiety Inventory score	8.3 ± 7.8	6.8 ± 5.1	*F*_(1,42)_ = 0.483	.491	0.011
POLYSOMNOGRAPHIC DATA					
Total sleep time (min)	476.9 ± 47.6	452.5 ± 32.5	*F*_(1,55)_ = 2.499	.120	0.042
Sleep onset latency (min)	7.2 ± 5.0	8.8 ± 10.0	*F*_(1,55)_ = 1.191	.280	0.021
REM sleep latency (min)	89.4 ± 43.3	90.6 ± 51.6	*F*_(1,55)_ = 0.161	.690	0.003
WASO (min)	31.6 ± 20.4	41.0 ± 22.0	*F*_(1,55)_ = 0.741	.393	0.012
Awakening index (nb./h)	2.7 ± 0.9	3.7 ± 1.7	*F*_(1,55)_ = 4.729	**.034**	0.077
Sleep efficiency (%)	93.7 ± 4.1	91.6 ± 4.5	*F*_(1,55)_ = 1.146	.286	0.018
N1 (%)	8.2 ± 4.0	11.1 ± 6.7	*F*_(1,55)_ = 2.695	.106	0.046
N2 (%)	58.3 ± 8.0	57.8 ± 6.4	*F*_(1,55)_ = 1.003	.321	0.016
N3 (%)	12.5 ± 7.0	12.4 ± 7.7	*F*_(1,55)_ = 0.787	.379	0.012
REM sleep (%)	20.2 ± 4.8	18.7 ± 3.0	*F*_(1,55)_ = 3.069	.085	0.053
Microarousal index	8.3 ± 4.3	9.4 ± 6.1	*F*_(1,55)_ = 0.252	.618	0.004
AHI (events/h)	1.7 ± 2.2	2.9 ± 3.8	*F*_(1,55)_ = 0.776	.382	0.013
PLMS index	6.7 ± 12.4	8.2 ± 10.9	*F*_(1,55)_ = 5.465e-4	.981	9.500e-6
Sleep stage transitions index (nb./h)	21.5 ± 5.0	23.5 ± 6.0	*F*_(1,55)_ = 0.710	.403	0.012
Transition REM to other stages (nb./h)	16.6 ± 7.2	18.3 ± 7.0	*F*_(1,55)_ = 0.706	.405	0.013
MSLT DATA					
Mean Total sleep time (min)	13.7 ± 2.1	12.9 ± 2.3	*F*_(1,56)_ = 0.547	.463	0.009
Mean Sleep onset latency (min)	4.7 ± 1.8	5.0 ± 1.7	*F*_(1,56)_ = 0.368	.546	0.007
Mean Sleep efficiency (%)	92.9 ± 5.7	88.0 ± 12.7	*F*_(1,56)_ = 1.425	.238	0.022
SOREMPs (nb.)	0.3 ± 0.5	0.1 ± 0.3	*F*_(1,56)_=3.349	.073	0.056

The results are expressed as the mean ± standard deviation for continuous variables, and as the number of participants and percentage for categorical variables. BMI: body mass index; ESS: Epworth Sleepiness Scale; PSG: polysomnography; AHI: apnea-hypopnea index; nb: number; NREM: non-REM sleep; PLMS: periodic legs movement in sleep; REM: rapid eye movement sleep; WASO: wake after sleep onset; SOREMPs: Sleep onset in rapid eye movement periods

**Table 3.** Demographic/clinical, polysomnographic and MSLT data of participants with subjective IH reporting unrefreshing vs. refreshing naps adjusted for age (when applicable)

	Unrefreshing naps (*n* = 33)	Refreshing naps (*n* = 20)	*F*(df) or χ^2^	*p*-value	Effect size or Cramer’s V
DEMOGRAPHIC/CLINICAL DATA					
Women (*n*; %)	23; 69.7%	12; 60.0%	χ^2^ = 0.522	.470	0.099
Age (years)	29.9 ± 6.7	34.5 ± 10.9	*F*_(1,46)_ = 0.246	.622	0.005
BMI (kg/m^2^)	23.5 ± 3.3	24.5 ± 4.5	*F*_(1,47)_ = 0.873	.355	0.018
Felt rested after the nighttime PSG (*n*; %)	4; 22.2%	6; 60.0%	χ^2^ = 3.996	**.046**	0.378
ESS score	16.3 ± 4.9	17.7 ± 2.7	*F*_(1,49)_ = 0.630	.431	0.012
Beck Depression Inventory score	9.6 ± 7.3	10.1 ± 6.0	*F*_(1,46)_ = 0.003	.959	5.705e-5
Beck Anxiety Inventory score	8.7 ± 9.0	9.0 ± 7.3	*F*_(1,46)_ = 0.020	.899	4.227e-4
POLYSOMNOGRAPHIC DATA					
Total sleep time (min)	445.3 ± 36.2	439.8 ± 40.8	*F*_(1,50)_ = 0.174	.678	0.003
Sleep onset latency (min)	13.7 ± 10.1	9.6 ± 6.0	*F*_(1,50)_ = 4.228	**.045**	0.074
REM sleep latency (min)	105.9 ± 48.3	92.5 ± 38.9	*F*_(1,50)_ = 0.750	.391	0.015
WASO (min)	44.5 ± 33.5	46.2 ± 30.4	*F*_(1,50)_ = 0.488	.488	0.008
Awakening index (nb./h)	3.2 ± 1.3	4.0 ± 1.5	*F*_(1,50)_ = 1.629	.208	0.026
Sleep efficiency (%)	91.0 ± 6.7	90.5 ± 6.5	*F*_(1,50)_ = 0.433	.514	0.007
N1 (%)	8.5 ± 3.4	12.7 ± 6.7	*F*_(1,50)_ = 5.345	**.025**	0.075
N2 (%)	54.5 ± 6.7	55.2 ± 5.0	*F*_(1,50)_ = 0.040	.842	7.091e-4
N3 (%)	15.8 ± 6.3	12.4 ± 7.8	*F*_(1,50)_ = 0.510	.479	0.006
REM sleep (%)	21.2 ± 4.2	19.7 ± 4.4	*F*_(1,50)_ = 1.297	.260	0.025
Microarousal index	8.1 ± 4.7	11.8 ± 5.8	*F*_(1,50)_ = 3.924	.053	0.067
AHI (events/h)	1.2 ± 1.9	2.4 ± 3.0	*F*_(1,50)_ = 1.813	.184	0.034
PLMS index	7.0 ± 11.0	11.1 ± 20.8	*F*_(1,50)_ = 0.039	.844	6.585e-4
Sleep stage transitions index (nb./h)	22.3 ± 5.3	27.0 ± 6.2	*F*_(1,50)_ = 6.069	**.017**	0.103
Transition REM to other stages (nb./h)	15.3 ± 6.0	16.2 ± 4.1	*F*_(1,50)_ = 0.043	.837	8.220e-4
MSLT DATA					
Mean Total sleep time (min)	9.1 ± 3.6	10.4 ± 3.1	*F*_(1,49)_ = 2.540	.117	0.048
Mean Sleep onset latency (min)	12.8 ± 2.8	12.1 ± 2.7	*F*_(1,49)_ = 1.429	.238	0.027
Mean Sleep efficiency (%)	80.1 ± 15.0	82.8 ± 17.3	*F*_(1,49)_ = 0.835	.365	0.016
SOREMPs (nb.)	0.4 ± 0.8	0.3 ± 0.6	*F*_(1,49)_ = 0.073	.788	0.001

The results are expressed as the mean ± standard deviation for continuous variables, and as the number of participants and percentage for categorical variables. BMI: body mass index; ESS: Epworth Sleepiness Scale; PSG: polysomnography; AHI: apnea-hypopnea index; nb: number; NREM: non-REM sleep; PLMS: periodic leg movement in sleep; REM: rapid-eye movement sleep; WASO: wake after sleep onset; SOREMPs: Sleep onset in rapid eye movement periods.

In the nighttime PSG, objective IH patients with unrefreshing naps had a lower awakening index compared to patients with refreshing naps.

In subjective IH, patients with unrefreshing naps had a lower percentage of N1 sleep and a lower sleep stage transitions index compared to the refreshing naps group. They also presented a longer sleep onset latency compared to patients with refreshing naps.

## Discussion

In this retrospective study, we found that IH participants reporting unrefreshing naps had more consolidated sleep, as reflected by a lower awakening index, a lower percentage of N1 sleep (particularly in the last third of the night), less sleep stage transitions, and a higher percentage of REM sleep compared to patients with refreshing naps. They were also younger, and more susceptible to report nonrestorative nighttime sleep in the morning after the PSG compared to those reporting refreshing naps. These differences in sleep architecture variables suggest that unrefreshing naps are not due to a poor sleep quality, but could be rather explained by a weak arousal drive. Our results are of particular interest, as self-reported unrefreshing naps may suggest a more severe disease that standard subjective (i.e. questionnaires) and objective (i.e. MSLT) EDS assessment protocols may not be able to capture.

### Refreshing vs. unrefreshing naps as a supportive IH clinical feature

The current sleep disorders classification integrates the unrefreshing aspect of naps as a supportive clinical feature for IH diagnosis [1]. There is, to date, a lack of studies investigating the clinical relevance of unrefreshing naps and whether or how patients reporting unrefreshing naps differ from those who report refreshing naps. Our data show that IH patients with and without refreshing naps do not differ in terms of demographic characteristics (with the exception of age), depression, anxiety, and sleepiness questionnaires or MSLT. The main group differences were on sleep fragmentation variables, where participants with unrefreshing naps had more consolidated sleep than participants with refreshing naps. Notably, patients with unrefreshing nighttime sleep did not have more consolidated sleep than those with refreshing nighttime sleep on the PSG recording. While previous studies have not investigated differences in clinical characteristics or sleep architecture in IH participants based on the refreshing quality of their naps, a previous study compared sleep architecture in patients with (*n* = 18) and without (*n* = 32) sleep drunkenness [9]. They found no group differences, except that participants with sleep drunkenness were more frequently of the evening chronotype as indicated by a lower Morningness–Eveningness Questionnaire score. This may explain why they possibly reported more drunkenness in the morning, while a possible circadian rhythm disorder cannot be excluded [15]. These results, combined with those from the present study, suggest that sleep drunkenness and the unrefreshing aspect of naps, but not that of nighttime sleep, are important clinical features.

Moreover, to date, many questions exist regarding the current objective criteria for IH [3]. The main electrophysiological criteria of IH diagnosis include a mean sleep onset latency of ≤ 8 min at the MSLT and/or a total sleep time ≥ 660 min measured by 24-hour PSG monitoring or by wrist actigraphy. Occasionally, patients do not meet these criteria and clinical judgment must be used to decide if these patients should be considered to have IH [1]. As a result, there is no official characterization of these two groups based on naps’ refreshness, to the best of our knowledge, and questions are still open regarding the different gradients of disease severity in these different groups. In our sample, participants with and without unrefreshing naps were equally distributed in the objective and subjective IH groups. Sensitivity analyses underlined that subjective IH patients presented more group differences (% of N1, sleep stage transition index) on PSG than objective patients (awakening index). Assessing the unrefreshing aspects of naps may be used as an additional marker of IH severity, especially when the MSLT is close to reaching pathological values or when clinical judgment is necessary to determine if these patients should be diagnosed with IH. Further studies should test whether including unrefreshing naps can help clinicians and researcher to distinguish IH subtypes.

### IH severity and phenotype subtypes

While IH patients may present a wide range of disease severity or phenotypic heterogeneity, current diagnosis criteria do not incorporate grades of severity or subtypes due to the lack of objective evidence. Clinicians often report that IH patients have a particularly good sleep quality, and current nosological criteria consider a high sleep efficiency (>90%) as an essential feature for IH diagnosis [1]. However, a systematic review and meta-analysis rather reported a reduced sleep efficiency in women with IH [16], suggesting possible subtypes based on sleep efficiency. The presence of hypersomnolence due to a psychiatric condition, which is more susceptible to be associated with lower sleep efficiency, could also explain part of these observations [17, 18]. In the present study, reporting unrefreshing naps was associated with markers of more consolidated sleep. In line with our results, other studies have reported that IH patients have a higher sleep efficiency and a lower sleep fragmentation index than healthy controls [8, 19]. This could point toward the existence of two distinct IH subgroups based on the unrefreshing aspect of naps and sleep consolidation. Whether presenting unrefreshing naps is associated with a more severe disease is unknown. Interestingly, unrefreshing naps were incorporated in the recently validated Idiopathic Hypersomnia Severity Scale [20], as well as in the Hypersomnia Severity Index questionnaire [21], which will provide interesting data for future studies.

### Arousal drive vs. sleep pressure

The pathophysiology of IH is incompletely understood. However, an excessive sleep drive may reduce the ability to maintain wakefulness in IH patients. While the classic sleep–wake switch model explains the possible abrupt awakenings through the lack of transitional states [22], recent studies have hypothesized the possible occurrence of intermediate stages [23]. According to this hypothesis, the desynchronized action of sleep pressure could keep patients with unrefreshing naps in an intermediate stage, making it difficult for them to fully awake from sleep and maintaining a state of constant sleepiness during the day. The lower quantity of N1 sleep occurring in the latter third of the night in participants with unrefreshing naps may further suggest a less progressive and harmonious transition out of sleep toward wakefulness.

The IH phenotype with unrefreshing naps could also represent a subgroup unable to efficiently reduce sleep pressure. However, according to a meta-analysis, IH patients have less slow-wave sleep compared to controls [16], which is the opposite pattern to what would be expected following sleep deprivation [24]. The results of the present study also showed no difference on slow-wave sleep between the refreshing and unrefreshing naps subgroups. Further studies should explore sleep pressure dissipation in large samples of IH patients.

### Sleep quality and overlap between IH and narcolepsy type 2

Sleep quality is a complex construct composed of both self-reported and objective aspects [25, 26]. Standard PSG parameters and quantitative electroencephalography measures have provided no definitive evidence of a possible objective indicator of sleep quality in healthy individuals [27]. However, factors such as sleep duration and continuity are more consistently associated with the subjective perception of sleep quality and are part of the definition of sleep health [28]. In the present study, there is a relationship between self-reported restorative nighttime sleep and refreshing aspects of naps. Indeed, IH subjects who reported refreshing naps during the day also reported refreshing nighttime sleep during the PSG. However, both groups (refreshing naps and unrefreshing naps) showed a high sleep efficiency (>90%) and a mean total sleep time of >7 h. Paradoxically, the refreshing naps subgroup also showed a higher sleep fragmentation index than the unrefreshing naps group. Whether presenting sleep inertia could explain the unrefreshing aspect of sleep despite a high-quality sleep based on PSG parameters is possible and further studies should investigate how sleep inertia upon awakening may be related to subjective sleep assessment. Interestingly, a treatment known for its ability to reduce fragmented night sleep, namely sodium oxybate, seems to improve both symptoms of narcolepsy and IH [29].

Importantly, refreshing daytime naps and sleep fragmentation are both clinical criteria for the diagnosis of narcolepsy type 2 (NT2). If our IH subjects did not strictly meet the current diagnostic criteria for NT2 (e.g. presence of > 2 SOREMPs), the diagnostic criteria of IH and NT2 are not specific and can overlap, explaining why it can be difficult to discriminate these two disorders [30, 31]. In line with these considerations, the refreshing naps group could represent an IH subgroup that would show some symptom overlapping with NT2. Recently, one study showed that a deficiency in the level of orexin could be considered a marker of nighttime sleep instability in hypersomnolence disorders (not only in NT1 but also in NT2 and IH) [32]. For these reasons, future studies should investigate orexin levels in relation to the refreshing aspects of naps in noncataplectic hypersomnia.

## Study limits

First, in the present study, most participants were assessed with sleep diaries, questionnaires, clinical interviews, and the PSG-MSLT protocol. Systematically using 7-day actigraphy or an extended 32-h ad libitum PSG protocol [18] would have allowed a better characterization of the participants sample by objectively measuring total sleep time. Second, due to the retrospective nature of our study, the refreshing and unrefreshing aspect of naps was assessed through clinical interviews rather than a standardized questionnaire. Third, the present study did not allow to verify whether the associations between the refreshing aspect of naps and the sleep parameters are specific to IH patients or could also be found in other healthy or clinical populations. As it is generally expected that restorative sleep is associated with better sleep quality, we may hypothesize that the associations observed are specific to the IH population. Fourth, it is possible that some IH patients may have both refreshing and unrefreshing naps without falling into a clear dichotomized category, and a scale providing a continuum for this assessment would be useful. For example, the Idiopathic Hypersomnia Severity Scale [20] offers a graded response choice for the question “In general, how do you feel after a nap? Very sleepy, sleepy, awake and wide awake?,” which is closely related to the refreshing aspect of nap. Fifth, we do not have in-depth information about the average number of naps during a day, their duration and the period of the day at which they occurred (i.e., morning, afternoon, evening). The use of validated scales and questionnaires is needed to better investigate naps and other clinical features of IH. Sixth, the MSLT procedure was performed with an adapted version with four naps while the current procedure recommends five naps. Finally, the evaluation of restorative/nonrestorative sleep during an in-laboratory PSG as compared to natural home condition could differ as sleep quality, during a first-night PSG, is generally worse than what is observed at home (first-night effect) [33, 34]. Importantly, in the ICSD-3, it is the usual refreshing and unrefreshing aspect of nap that is mentioned as an important clinical feature and in the present study, we found that this particular clinical characteristic is associated with differences in PSG parameters. The present study brings new interesting findings regarding the PSG characteristics associated with unrefreshing naps that may encourage future studies with standardized experimental protocols.

## Conclusions

Our study shows that self-reported unrefreshing naps are associated with more consolidated sleep in IH patients. Whether patients with unrefreshing naps have a more severe disease is currently unknown, but the refreshing aspects of naps should be more systematically assessed in clinical interviews. Using standardized hypersomnia severity scales will significantly improve data collection, but a standardized questionnaire on naps could also be developed. Given the multifactorial nature of IH, in-depth sleep microarchitecture studies are needed to better understand subgroup differences in IH patients. Furthermore, a more detailed and standardized analysis of naps could allow to better characterize patient subgroups and deepen our understanding of the IH pathophysiology.

## Supplementary Material

zsad175_suppl_Supplementary_Material

## Notes

**Financial disclosure:** This research project was supported by a Research Strategic Award from the American Academy of Sleep Medicine Foundation (Grant 227-SR-20).. N.G. holds a Canada Research Chair in Sleep disorders and brain health. A-S.D-R. holds a Canada Graduate Scholarships Doctoral Research Award (CGS-D) from the Canadian Institutes of Health Research.

**Non-financial disclosure:** None of the authors report conflicts of interest related to this article

**Data Availability Statement**: The data underlying this article will be shared on reasonable request to the corresponding author.
