# Supplementary material for: Are unrefreshing naps associated with nocturnal sleep architecture specificities in idiopathic hypersomnia?
Source: Sleep. 2023 Jul 1;46(11):zsad175. doi: 10.1093/sleep/zsad175 (PMC10636245; doi:10.1093/sleep/zsad175)
Supplement: zsad175_suppl_Supplementary_Material [file zsad175_suppl_supplementary_material.docx]

Are unrefreshing naps associated with nocturnal sleep architecture specificities in idiopathic hypersomnia?

Samantha Mombelli^1,2^, Anne-Sophie Deshaies-Rugama^1,3^, Hélène Blais,^1^ Zoran Sekerovic^1^, Cynthia Thompson^1^, Alex Desautels^1,4^, Jacques Montplaisir^1,2^, Milan Nigam^1,4^, Julie Carrier^1,3^, Nadia Gosselin^1,3^

1 Center for Advanced Research in Sleep Medicine, Research center of the Centre intégré universitaire de santé et de services sociaux du Nord de l’Île-de-Montréal, Montréal, Canada

2 Department of Psychiatry and Addictology, Université de Montréal, Montréal, Canada

3 Department of Psychology, Université de Montréal, Montréal, Canada

4 Department of Neuroscience, Université de Montréal, Montréal, Canada

Corresponding author:

Nadia Gosselin, Ph.D.

Center for Advanced Research in Sleep Medicine

Hôpital du Sacré-Cœur de Montréal

5400 boul. Gouin Ouest, office J-5135, Montréal, Québec, H4J 1C5, Canada

Tel: 514-338-2222 ext. 7717; Fax: 514-338-3893

Email: nadia.gosselin@umontreal.ca

**Supplementary Material**

**S 1.** Demographic/clinical, polysomnographic and MSLT data of patients presenting objective markers of IH (objective IH) vs. IH patients diagnosed based on clinical features (subjective IH) adjusted for age (when applicable)

|  | Subjective IH  (n=53) | Objective IH  (n=59) | F(df) or $\boldsymbol{\chi}$^2^ | p-value | Effect size or Cramer’s V |
| --- | --- | --- | --- | --- | --- |
| DEMOGRAPHIC/CLINICAL DATA |  |  |  |  |  |
| Women (n; %) | 35; 66.0% | 41; 69.5% | $\boldsymbol{\chi}$^2^=0.153 | 0.696 | 0.037 |
| Age (years) | 31.6 ± 8.7 | 36.2 ± 10.8 | F_(1,110)_=5.841 | **0.017** | 0.050 |
| BMI (kg/m^2^) | 23.9 ± 3.8 | 25.1 ± 4.4 | F_(1,99)_=1.499 | 0.224 | 0.015 |
| Felt rested after the nighttime PSG (n; %) | 10; 35.7% | 9; 26.5% | $\boldsymbol{\chi}$^2^=0.617 | 0.432 | 0.100 |
| ESS score | 16.8 ± 3.6 | 17.0 ± 3.7 | F_(1,104)_=0.071 | 0.790 | 6.634e-4 |
| Beck Depression Inventory score | 9.8 ± 6.8 | 11.5 ± 9.1 | F_(1,91)_=0.815 | 0.369 | 0.009 |
| Beck Anxiety Inventory score | 8.8 ± 8.3 | 7.7 ± 6.8 | F_(1,91)_=0.708 | 0.402 | 0.008 |
| POLYSOMNOGRAPHIC DATA |  |  |  |  |  |
| Total sleep time (min) | 443.2 ± 37.7 | 467.2 ± 43.6 | F_(1,108)_=16.384 | **<.001** | 0.117 |
| Sleep onset latency (min) | 12.1 ± 8.9 | 7.8 ± 7.4 | F_(1,108)_=7.801 | **0.006** | 0.067 |
| REM sleep latency (min) | 100.8 ± 45.1 | 89.9 ± 46.3 | F_(1,108)_=0.934 | 0.336 | 0.008 |
| WASO (min) | 45.1 ± 32.1 | 35.3 ± 21.4 | F_(1,108)_=8.820 | **0.004** | 0.065 |
| Awakening index (nb./h) | 3.5 ± 1.5 | 3.1 ± 1.3 | F_(1,108)_=5.347 | **0.023** | 0.042 |
| Sleep efficiency (%) | 90.8 ± 6.5 | 92.9 ± 4.4 | F_(1,108)_=10.043 | **0.002** | 0.072 |
| N1 (%) | 10.1 ± 5.3 | 9.3 ± 5.4 | F_(1,108)_=2.770 | 0.099 | 0.022 |
| N2 (%) | 54.7 ± 5.8 | 58.1 ± 7.3 | F_(1,108)_=4.214 | **0.043** | 0.034 |
| N3 (%) | 14.6 ± 7.0 | 12.5 ± 7.2 | F_(1,108)_=0.233 | 0.630 | 0.002 |
| REM sleep (%) | 20.6 ± 4.3 | 20.1 ± 4.3 | F_(1,108)_=0.166 | 0.685 | 0.002 |
| Microarousal index | 9.5 ± 5.4 | 8.8 ± 5.0 | F_(1,108)_=1.749 | 0.189 | 0.015 |
| AHI (events/h) | 1.7 ± 2.4 | 2.1 ± 3.0 | F_(1,108)_=0.058 | 0.811 | 4.918e-4 |
| PLMS index | 8.5 ± 15.4 | 7.3 ± 11.7 | F_(1,108)_=1.474 | 0.227 | 0.012 |
| Sleep stage transitions index (nb./h) | 24.0 ± 6.0 | 22.3 ± 5.4 | F_(1,108)_=5.351 | **0.023** | 0.044 |
| Transition REM to other stages (nb./h) | 15.6 ± 5.3 | 17.3 ± 7.1 | F_(1,108)_=1.334 | 0.251 | 0.012 |
| MSLT DATA |  |  |  |  |  |
| Mean total sleep time (min) | 9.6 ± 3.4 | 13.3 ± 2.2 | F_(1,108)_=53.730 | **<.001** | 0.324 |
| Mean sleep onset latency (min) | 12.4 ± 2.7 | 4.8 ± 1.8 | F_(1,108)_=303.840 | **<.001** | 0.736 |
| Mean sleep efficiency (%) | 81.2 ± 15.8 | 90.9 ± 9.4 | F_(1,108)_=20.965 | **<.001** | 0.155 |
| SOREMPs (nb.) | 0.3 ± 0.7 | 0.3 ± 0.4 | F_(1,108)_=0.167 | 0.684 | 0.001 |

The results are expressed as the mean ± standard deviation for continuous variables, and as the number of participants and percentage for categorical variables. BMI: body mass index; ESS: Epworth Sleepiness Scale; PSG: polysomnography; AHI: apnea-hypopnea index; nb.: number; IH: idiopathic hypersomnia; MSLT: Mean sleep onset latency; NREM: non-REM sleep; PLMS: periodic leg movement in sleep; REM: rapid-eye movement sleep; WASO: wake after sleep onset**;** SOREMPs: sleep onset in rapid eye movement period
